# Supplementary material for: Biocompatible designated Resin-3D-printed polymers exhibit reproductive toxicity prevented by Parylene-C
Source: bioRxiv. 2026 Jun 10:2026.06.05.730268. Preprint. [Version 1] doi: 10.64898/2026.06.05.730268 (PMC13278130; doi:10.64898/2026.06.05.730268)

bioRxiv preprint doi: https://doi.org/10.64898/2026.06.05.730268; this version posted June 10, 2026. The copyright holder for this preprint (which was not certified by peer review) is the author/funder, who has granted bioRxiv a license to display the preprint in perpetuity. It is made available under a CC-BY 4.0 International license.

Supporting Information

Biocompatible designated Resin-3D-printed polymers exhibit reproductive toxicity prevented by Parylene-C

*Hannes Campo[1], Uyen Tran[1], Yiru Zhu[1], Hoi Chang Lee[1], and Francesca E. Duncan[1,2]**

[1]Department of Obstetrics and Gynecology, Feinberg School of Medicine, Northwestern University, Chicago, IL, 60611, [2]Buck Institute for Research on Aging, Novato, CA, 94945.

Table of contents entry:

New approach methodologies such as microphysiological systems (MPS) are designed to reduce reliance on animal models for basic and clinical research. Leachates from resin-3D-printed components represent a risk to incorporating 3D-printed components into MPS. MEIOSA is a Multi-Endpoint Oocyte Safety Assay that stratifies biocompatibility of ISO 10993 compliant resins and demonstrates the ability of Parylene-C to rescue cytotoxicity.

Figure S1. Effect of aging and overcuring protocols on BioMed Clear. A) Representative images of oocytes observed after IVM (left, scale bar: 100 μm) with primary phenotypes (right, scale bar: 10 μm) for BM Clear at different timepoints post-curing. B) Resulting meiotic progression incidence for BM Clear at different timepoints post-curing. For each timepoint 30 oocytes were used. C) Meiotic progression incidence for BM Durable and Clear V4 at different timepoints post-curing. For each timepoint 30 oocytes were used. D) Representative images of primary phenotype observed after IVM for BM Clear after overcure protocol 1 and 2 (OCP). OCP 1 and OCP 2 conditions were tested using 15 and 60 oocytes, respectively. E) Resulting meiotic progression incidence for BM Clear after OCP 1 and 2 compared to control. F) Representative z-projections of MII eggs. Actin (rhodamine phalloidin, red), α-tubulin (green), and DNA (DAPI, blue) were detected by immunocytochemistry. PB indicated by triangle, scale bar: 10 μm. G) Incidence of normal and aberrant chromosome alignment for aged and OCP 1 and 2 treated BM Clear.

Figure S2. Effect of Parylene-C coating procedure on insert dimensions. A) Width measurements of fully cured inserts for each resin type. Compact letter display (A, B, C, D) shows a significance of 0.05 or less between resins (n=54 for Clear v4, BM Clear, BM Durable and n=56 for BM 50A and BM 80A). B) Normalized width after curing and after coating shows differential effect of the coating procedure. * represents $P < 0.05$.

bioRxiv preprint doi: https://doi.org/10.64898/2026.06.05.730268; this version posted June 10, 2026. The copyright holder for this preprint (which was not certified by peer review) is the author/funder, who has granted bioRxiv a license to display the preprint in perpetuity. It is made available under a CC-BY 4.0 International license.

Figure S3. Developmental parameters of embryo derived from *in vivo* obtained MII oocyte. A) Representative timelapse images with 24-hour increments for embryos that reached blastocyst stage. B) Morphokinetic timepoint analysis of embryo development (n=2, 16 embryos). C) Quantification of time required to reach 2-cell, 4-cell, 8-cell, and early blastocyst stages (eBL) of development of healthy MII oocytes from *in vivo* mice (n=2, 16 embryos). D) Calculation of time between successive stages. E) Representative z-projections of day-5 embryo stained with actin (rhodamine phalloidin, red), α-tubulin (green), and DNA (DAPI, blue) by immunocytochemistry. F) Quantification of DAPI-positive nuclei counts (n=20). G) Mitotic index percentage of day-5 embryos (n=18).

Supplementary Video S1. Representative timelapse video of embryo derived from *in vivo* obtained MII oocyte.

Supplementary Video S2. Representative timelapse video of embryo derived from MII oocyte matured *in vitro*.

Supplementary Video S3. Representative timelapse video of embryo derived from MII oocyte matured *in vitro* with Parylene-C coated BioMed Flex 80A.

(which was not certified by peer review) is the author/funder, who has granted bioRxiv a license to display the preprint in perpetuity. It is m
available under a CC-BY 4.0 International license.

Supplemental Figure 1.

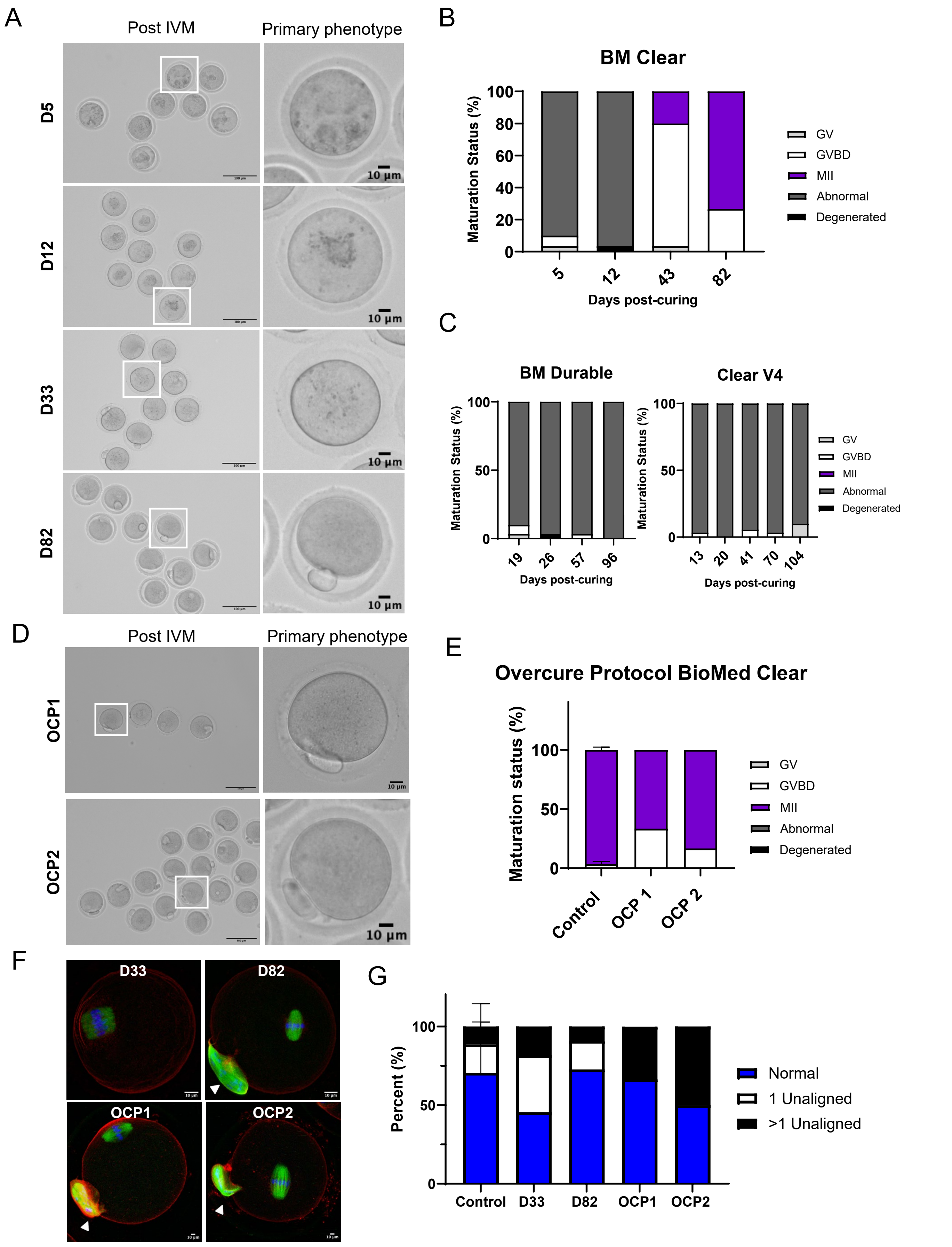

(which was not certified by peer review) is the author/funder, who has granted bioRxiv a license to display the preprint in perpetuity. It is m
available under a CC-BY 4.0 International license.

Supplemental Figure 2.

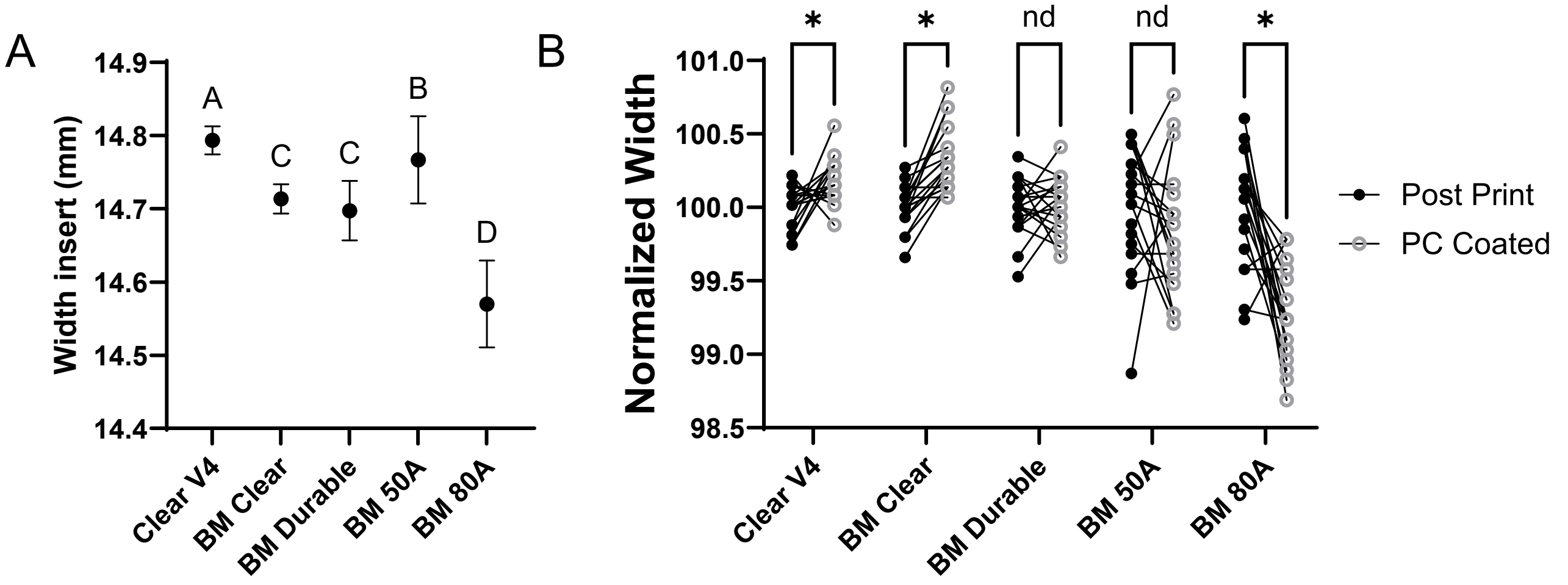

(which was not certified by peer review) is the author/funder, who has granted bioRxiv a license to display the preprint in perpetuity. It is m
available under a CC-BY 4.0 International license.

Supplemental Figure 3.

A

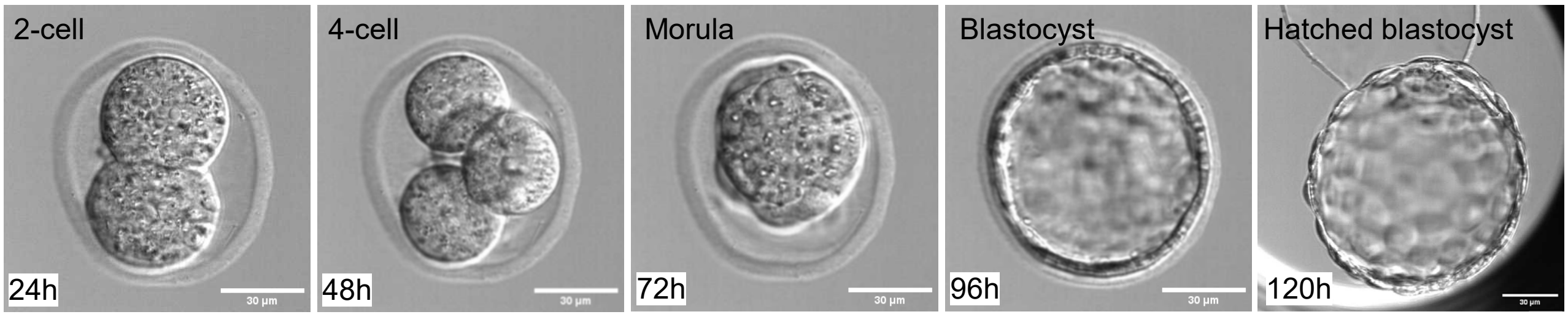

B

	Time to (h):	2-cell	4-cell	8-cell	Blastocyst
Treatment	In Vivo Control	17.7 ± 1.0	40.1 ± 2.1	54.65 ± 2.6	85.65 ± 8.0

C D E F G

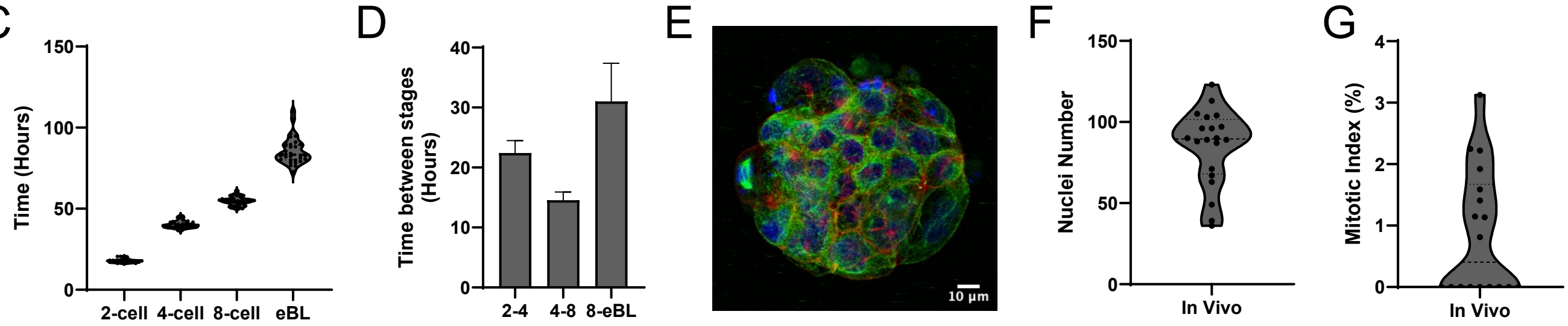

Supplement: Supplementary file 1 [file NIHPP2026.06.05.730268v1-supplement-4.pdf]
